# Implementing traumatic brain injury screening in behavioral healthcare: protocol for a prospective mixed methods study

**DOI:** 10.1186/s43058-022-00261-x

**Published:** 2022-02-14

**Authors:** Kathryn A. Coxe-Hyzak, Alicia C. Bunger, Jennifer Bogner, Alan K. Davis, John D. Corrigan

**Affiliations:** 1 College of Social Work, The Ohio State University, 1947 College Road N, Columbus, OH 43210 USA; 2 Department of Physical Medicine and Rehabilitation, The Ohio State University College of Medicine, Columbus, OH USA; 3 Center for Psychedelic and Consciousness Research, Department of Psychiatry and Behavioral Sciences, Johns Hopkins University, Baltimore, MD USA; 4 Department of Psychiatry, College of Medicine, The Ohio State University, Columbus, OH USA; 5 Ohio Valley Center for Brain Injury Prevention and Rehabilitation, The Ohio State University Wexner Medical Center, Columbus, OH USA

**Keywords:** Traumatic brain injury, OSU TBI-ID, TBI screening, Behavioral health treatment, Mixed methods, Theory of planned behavior, Diffusion of innovations, CFIR, Acceptability, Feasibility

## Abstract

**Background:**

Characteristics of both individuals and innovations are foundational determinants to the adoption of evidenced-based practices (EBPs). However, our understanding about what drives EBP adoption is limited by few studies examining relationships among implementation determinants and implementation outcomes through theory-driven hypothesis testing. Therefore, drawing on the Theory of Planned Behavior and Diffusion of Innovations Theory, this study will disentangle relationships between provider characteristics and innovation factors on the early adoption of the Ohio State University Traumatic Brain Injury Identification Method (OSU TBI-ID) in behavioral health settings.

**Methods:**

This study will utilize an explanatory sequential mixed methods design. In Phase I (quantitative), Time 1, we will investigate behavioral health providers (*N* = 200) attitudes, perceived behavioral control, subjective norms, and intentions to screen for TBI upon completion of a video module introducing the OSU TBI-ID. At Time 2, we will examine the number of TBI screens conducted over the previous month, as well as the feasibility, appropriateness, and acceptability of using the OSU TBI-ID in practice. Structural equation modeling will be used to determine whether provider characteristics predict TBI screening intentions, and whether intentions mediate actual TBI screening behaviors. We will then test whether feasibility, appropriateness, and acceptability of the OSU TBI-ID moderates the relationship between intentions and TBI screening behaviors. In Phase II (qualitative), we will develop an interview guide using results from Phase I and will conduct semi-structured interviews with providers (*N* = 20) to assess contextual determinants of TBI screening adoption. Qualitative data will be thematically analyzed using sensitizing concepts from the Consolidated Framework for Implementation Research and integrated with the quantitative results using a joint display.

**Discussion:**

This mixed methods study capitalizes on two theory-driven hypotheses bridging proximal (e.g., screening intent) to distal (actual behaviors) implementation outcomes and will contextualize these results qualitatively to advance our understanding about why TBI screening adoption has failed to translate to the behavioral healthcare context. Results of this study will offer insights into what is driving TBI screening adoption so that implementation strategies can be selected with greater precision to improve the adoption, sustainment, and scale-up of TBI screening in behavioral healthcare.

**Supplementary Information:**

The online version contains supplementary material available at 10.1186/s43058-022-00261-x.

Contributions to the literature

- How provider and intervention characteristics interact to affect evidenced-based practice (EBP) adoption in behavioral healthcare remains unclear, leaving gaps in our understanding about the key drivers to EBP adoption.
- This mixed methods study (1) investigates the contribution of behavioral health providers’ attitudes, subjective norms, and perceived behavioral control to the relationship between TBI screening intention and behaviors, (2) examines whether acceptability, feasibility, and appropriateness of TBI screening moderate the relationship between intention and behaviors, and (3) contextualizes TBI screening adoption through interviews with providers.
- Results will facilitate implementation strategy selection tailored to these contexts to increase TBI screening adoption.

## Background

Multiple challenges prohibit the implementation of evidence-based practice (EBP) innovations in behavioral healthcare settings. Implementation research points to the characteristics and behaviors of clinicians acting within the treatment context as primary influencers in the adoption of EBPs, particularly during early phases of implementation [1, 2]. Provider characteristics, including attitudes toward an innovation or perceived control over implementing the innovation are well-known determinants (e.g., barriers and facilitators) affecting EBP adoption [2]. Other known determinants affecting implementation adoption are factors related to the innovation itself, which may include innovation complexity, appropriateness of the innovation to the specific context or treatment practices, feasibility of implementing the innovation within that context, and perceived acceptability of the innovation [1, 3]. Yet, these determinants do not likely operate individually during early phases of implementation, but rather as a function of the other on EBP adoption within the realm of the service context [1, 4]. For instance, although providers may consider the intervention appropriate (i.e., relevant to their client population), and they may report strong intentions to implement the innovation, their actual behaviors may not reflect any practice changes because the intervention was not feasible to conduct in practice. Subsequently, adoption of the innovation may be low, and therefore the desired practice remains unchanged. However, interactions between provider characteristics, innovation factors, and implementation outcomes are not well-studied, leaving the relationships between these interactions and under what context these interactions materialize unclear [5].

Although determinants are foundational to the success or failure of EBP adoption, our understanding about the key drivers influencing EBP adoption and service integration in the behavioral health environment can be improved through better precision and specification of relationships among constructs through theory-driven hypothesis testing [3, 6–9]. Numerous implementation frameworks identify and define multi-level constructs important to innovation adoption [1, 10, 11], but relationships between these constructs are rarely specified [7], leaving researchers and clinicians to wade through muddled processes in their attempt to understand why innovation adoption in behavioral health contexts has failed or succeeded. Differentiation between the implementation determinants from the mediators and moderators acting as drivers to EBP adoption using theory-driven hypotheses can help us to target where along the implementation cascade EBP adoption occurs [3, 6, 7]. Lack of or misspecification among determinants, mediators, and moderators may not only restrict our understanding of the implementation process, but also inhibits our ability to select appropriate implementation strategies that target these modifiers [6]. Using theoretically driven hypotheses can offer insight into what constructs to test, how to specify them, and where they should be placed along the implementation cascade (proximally or distally) [3, 9] so that implementation strategies can be appropriately selected [5, 12].

Further compounding this problem, however, is the lack of implementation studies addressing the treatment integration of services for clients with complex physical and mental health comorbidities into behavioral health contexts, thereby leaving gaps in our understanding about why EBP innovations developed for these clients have failed to penetrate this service landscape. Although studies in traumatic brain injury (TBI) screening and assessment have demonstrated improved symptom delineation between mental health and neuropsychological symptoms leading to mental health referrals [13] and that validated TBI screening methods can improve clinical care decisions [14], the processes that increase the adoption of these services in behavioral health settings is unknown. Therefore, integrated care pathways that involve complex interventions with multiple components (e.g., screening, intervention adaptation, referral) [15] for these individuals are inconsistent or absent and we are left to question why these clients do not experience more successful outcomes.

### Research-to-practice gap: implementing TBI screening in behavioral healthcare settings as a first step toward optimizing care

An estimated 50% of clients seeking treatment for substance use or other mental health comorbidities have a lifetime history of TBI [16]. TBI is a complex health condition and a leading cause of disability among adults in the United States [17]. A TBI is a type of acquired brain injury that occurs when an object forcefully hits the head, the head hits an object, or an object pierces the skull and enters brain tissue [18]. A TBI may also result from whiplash effects or blast-induced head trauma. Persistent neurological changes to brain structure and function [19] elevate the acute injury to a chronic, dynamic process affecting physical, psychological, and social domains over the lifespan [20]. Studies demonstrate that TBI can lead to the onset of new or worsened risky substance use and/or mental health conditions, such as anxiety and depression, as well as higher likelihood of mental health service utilization or psychiatric hospitalization in later life [21–23]. TBI of any severity (e.g., concussions/mild, moderate, or severe) can also lead to cognitive dysfunction (e.g., learning and memory deficits, poor comprehension) [24, 25], maladaptive social behaviors (e.g., poor social awareness), behavioral dysregulation (e.g., aggression, emotional outbursts) [26], and poor coping skills [18, 27–29]. These changes can create challenges to clients’ ability to fully engage in and benefit from behavioral health treatment. Behavioral health treatment approaches should be adapted to accommodate client need (e.g., shortened treatment session or frequent reminders about appointments), but this first requires implementation of systematic screening methods to identify which clients need adapted behavioral health treatment [19, 20].

TBI is often underrecognized by providers in behavioral health treatment settings due to lack of provider or client awareness of TBI, as well as lack of provider skills and self-efficacy to screen for lifetime history of TBI using established screening methods [14, 30]. Lack of TBI identification in adults with risky substance use or mental health comorbidities may result in misattribution of the symptoms of TBI leading to mislabeling the client as ‘non-compliant’ or poorly motivated [31], and could affect treatment or intervention decisions that do not account for TBI-related sequela.

### The evidence-based practice innovation

The Ohio State University TBI Identification method (OSU TBI-ID) is a comprehensive, evidence-based TBI screening method that behavioral health providers can use to screen for lifetime history of TBI in 3–5 min and was first validated among a cohort of clients seeking substance use disorder treatment in behavioral health settings [22, 23]. The OSU TBI-ID uses optimal recall methods to prompt a client’s recall of injuries to the head and neck, then determines whether each injury was a TBI. Multiple indices of the extent of one’s lifetime history of TBI are derived [32] including age at first injury, worst injury (based on length of loss of consciousness), most recent injury (moderate or severe injuries in recent months or any TBI in recent weeks), multiple injuries, and TBI from repeated impacts like blasts experienced in combat or blows to the head incurred from domestic violence [33, 34]. Ascertaining lifetime exposure to TBI through structured elicitation of self-report is superior to relying only on medical record data, single questions about TBI (i.e., “Have you ever sustained a TBI?”), or assessment procedures like neuroimaging or neuropsychological assessment that are specific but not sensitive to a person’s exposure to TBI in their entire life. Self-report is particularly advantageous for lifetime TBI identification among vulnerable populations who may not have sought treatment for their injuries. Despite the current use of the OSU TBI-ID in other health, community, and rehabilitation settings [9, 25, 26], this TBI screening method has not been widely adopted in behavioral health treatment.

This protocol describes a prospective, mixed-methods study that investigates early adoption of the OSU TBI-ID in behavioral healthcare settings. We seek to understand providers’ attitudes and beliefs about adopting the OSU TBI-ID into routine service delivery, as well as the acceptability, feasibility, and appropriateness of implementing the OSU TBI-ID into the behavioral health service context.

### Theoretical foundations

This study is driven by the Theory of Planned Behavior (TPB) [35] and Roger’s Diffusion of Innovations Theory (DOI) [36, 37] due to the salience of provider characteristics and factors related to the innovation in predicting the early adoption of new innovations in behavioral healthcare settings [1, 38]. The TPB specifies provider-level characteristics (i.e., attitudes, perceived behavioral control, and subjective norms) that are known to influence providers’ intentions to adopt innovations in behavioral healthcare settings [35]. Specifically, provider attitudes toward TBI screening, perceived control over screening, and the perceived social pressures to screen for TBI may influence providers’ intentions to adopt the OSU TBI-ID into service delivery. Previous studies have used the TBP to examine providers’ intentions to adopt TBI-specific interventions in other health settings [39]. However, studies have yet to examine how these provider-level characteristics influence TBI screening intention and actual TBI screening behaviors in behavioral healthcare settings where many individuals with comorbid TBI, risky substance use, and mental health conditions seek treatment.

Roger’s Diffusion of Innovations Theory suggests that innovation-level factors (i.e., acceptability, feasibility, and appropriateness) are also critical components to the adoption of innovations in behavioral healthcare [1, 37]. Though acceptability, feasibility, and appropriateness of the innovation are often examined as implementation outcomes [3], these factors could potentially be the moderators that bridge providers’ intent to screen for TBI to actual TBI screening behaviors. Whereas the TPB specifies provider characteristics as determinants to intentions and ultimately TBI screening behaviors, the extent to which the relationship between intentions (proximal indicator) and the level of TBI screening adoption (distal outcome) may be moderated by factors related to the innovation. Specifically, the extent to which the OSU TBI-ID is perceived as acceptable, feasible, and relevant to the service landscape (i.e., client need, within the scope of practice) could influence whether TBI screening is used and to what extent. For instance, although positive attitudes toward TBI screening may predict stronger intentions to conduct TBI screening and ultimately TBI screening behaviors, providers may find that the innovation does not fit within the current service context which therefore reduces the extent to which TBI screening is used. Similarly, while providers may report high levels of control over their ability to implement the innovation and therefore stronger intention to perform the innovation, actual implementation of the innovation may be thwarted by feasibility concerns once providers have had the chance to trial the innovation, thereby affecting overall adoption of that innovation in practice. Testing the relationships between intention (proximal indicator), moderators (acceptability, feasibility, and appropriateness), and TBI screening behaviors (distal outcome) could improve our knowledge and understanding about why TBI screening has failed to translate to the behavioral health service context. Subsequently, implementation strategies that directly target these moderators can be developed, tested, and used in other behavioral healthcare settings where EBP adoption and integration of services for complex health conditions is lacking.

#### Service context

Assessing the contextual determinants to TBI screening is also vital to identifying implementation strategies aimed to increase the uptake and sustainment of TBI screening among behavioral health providers [40]. Assessing the implementation context and environment under which a new innovation is used is necessary to connecting hypotheses and theorized mechanisms to behaviors [40]. Assessing the service context adds depth to our understanding of how and why TBI screening is or is not adopted in behavioral health settings [40, 41]. Based on the context, implementation strategies can be identified that harness a social environment that is supportive of the innovation.

### Study aims

**Fig. 1:**
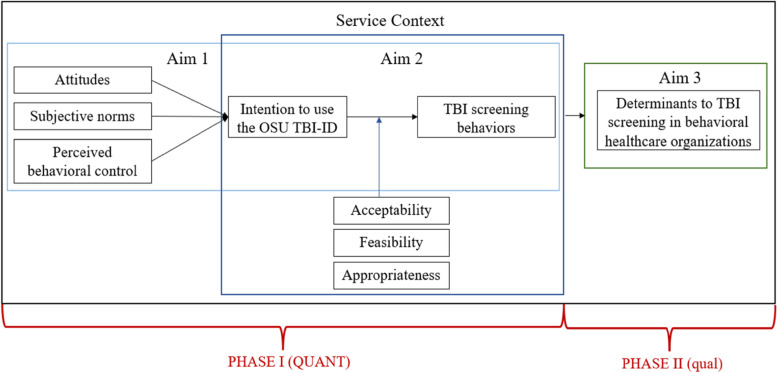
Conceptual model

This study will investigate the adoption of TBI screening in behavioral health settings through three specific aims. Figure 1 provides the conceptual model specifying each aim and relationships among constructs.

**Aim 1:** Examine the relationships between behavioral health providers’ attitudes, perceived behavioral control, and subjective norms as predictors to TBI screening intentions and examine whether intentions to adopt TBI screening mediate actual TBI screening behaviors at a one-month follow-up.

*Hypothesis 1:* Providers who have more favorable attitudes, greater perceived behavioral control, and greater perceived social pressure within the organization to screen for TBI will demonstrate greater TBI screening intentions and will report higher TBI screening behaviors at the one-month follow-up.

**Aim 2:** Investigate whether the acceptability, feasibility, and appropriateness of TBI screening using the OSU TBI-ID moderates the relationship between TBI screening intentions and actual TBI screening behaviors.

*Hypothesis 2:* Greater perceived acceptability, feasibility, and appropriateness of TBI screening using the OSU TBI-ID will strengthen the relationship between TBI screening intent and actual TBI screening behaviors.

**Aim 3:** Assess the contextual determinants to TBI screening adoption. We will investigate determinants to TBI screening adoption through qualitative, semi-structured interviews with a subset of behavioral health providers who completed the quantitative surveys.

## Methods/design

### Design overview

We use the Journal Article Reporting Standards for Mixed Methods Research (JARS-MMR) for reporting on all components of this mixed methods study throughout this article (see Additional File 1: Appendix) [42]. This study will utilize an explanatory sequential mixed methods design to prospectively investigate the provider-level characteristics, innovation-level factors, and contextual determinants to early TBI screening adoption in behavioral healthcare settings. The explanatory sequential mixed methods design consists of two distinct, consecutive phases, where emphasis is placed on the quantitative phase and the qualitative phase is used to contextualize the quantitative results (QUANT ➔ qual) [43, 44]. The mixed methods approach in this study will include comprehensive data collection and analytical techniques to combine theory-driven hypothesis-testing with in-depth assessments that explain the implementation of TBI screening in behavioral healthcare. Utilizing comprehensive data collection methods could help us understand the complex interplay of this environment with the innovation, which is particularly important to our understanding about service integration for individuals with physical and mental comorbidities.

Phase I (QUANT) will focus on theory-driven hypotheses to test the relationships among provider-level characteristics and innovation-level factors hypothesized to affect TBI screening adoption in behavioral healthcare settings. We will prospectively investigate behavioral health providers’ attitudes, perceived behavioral control, subjective norms, and intentions to adopt this TBI screening method into service delivery (Aim 1). After 1 month following completion of the survey, providers will receive a second survey assessing the proportion of TBI screens conducted over the past month, as well as the perceived acceptability, feasibility, and appropriateness of using the OSU TBI-ID in practice (Aim 2). Using the two-time point approach, this study will investigate theory-driven relationships between provider-level characteristics and innovation-level factors by capitalizing on theory-driven hypotheses to bridge proximal (e.g., screening intent) to distal (actual behaviors) outcomes with better precision. The expected outcome of Phase I is to identify mediators and moderators to inform our understanding of the conditions under which TBI screening adoption occurs.

Phase II (qual) will build upon the quantitative results through qualitative interviews with behavioral health providers to assess any additional determinants within the behavioral healthcare context affecting TBI screening adoption [40, 45]. We will develop a qualitative interview guide using the quantitative results from Phase I. Recognizing that additional determinants beyond characteristics of providers and factors related to the TBI screening innovation trigger influences to EBP adoption, and that behavioral health organizations inherently differ by type and resources (e.g., community-based, hospital-based, private practice) we will purposively recruit and interview a subset of providers from Phase I to assess the contextual determinants to the adoption of the OSU TBI-ID into service delivery (Aim 3). The qualitative results will expand on or dispute the results from our hypotheses by contextualizing TBI screening adoption within this behavioral health service landscape.

### Participants and setting

Participants will be behavioral health providers (*N* = 200) employed in behavioral health settings throughout the United States (e.g., private practices, community-based mental health clinics). Providers will be identified through national organizations and directories of behavioral health providers. To be eligible for this study, participants must be 18 years and older, English speaking, and currently employed as a licensed behavioral health provider in the United States (e.g., Licensed Psychologists, Clinical Social Workers, Professional Clinical Counselors, Professional Counselors, Marriage and Family Therapists, and/or Chemical Dependency Counselors).

### PHASE I (QUANT)

#### Aim 1

 Examine the relationships between behavioral health providers’ attitudes, perceived behavioral control, and subjective norms as predictors to TBI screening intentions and examine whether intentions to adopt TBI screening mediate actual TBI screening behaviors at a one-month follow-up.

#### Aim 2

 Investigate whether the acceptability, feasibility, and appropriateness of TBI screening using the OSU TBI-ID moderates the relationship between TBI screening intentions and actual TBI screening behaviors.

#### Procedures

##### Recruitment and data collection

Phase I data collection will consist of two consecutive time points using a prospective cohort study design. At Time 1, providers nationally will be emailed a detailed description about the study, the study inclusion criteria, and the Qualtrics link containing the OSU TBI-ID video module and Time 1 survey measures (Aim 1). Consent to participate will be on the first page of the Qualtrics survey, where proceeding to the survey questions signifies informed consent. Providers will first be asked to watch the 30-min, OSU TBI-ID video module to raise awareness about why TBI screening is relevant to behavioral health treatment, introduce the OSU TBI-ID screening form, and demonstrate step-by-step procedures on how to administer the OSU TBI-ID screening method using case exemplars. Providers will then be asked about their attitudes toward using this screening method, perceived social pressures to use the screening method, perceived control over using the screening method, and their intentions to use this screening method over the next month. The total time to complete the Time 1 survey is approximately 15 min. Providers will receive a Certificate of Completion to submit for 1 free continuing education unit and will be entered into a raffle for the chance to win a $50 gift card from a list of university-approved vendors. A total of 60 winners will be selected using a random number generator in Excel.

At Time 2, providers will be sent a second survey one-month later that assesses their perceptions of the acceptability, feasibility, and appropriateness of using the OSU TBI-ID screening method in practice after they have had the chance to trial the intervention. The survey will also ask them to report the number of times they used this screening method during the 1 month since watching the training video (Aim 2). To increase the response rate for Time 2, the Dillman method will be applied [46]. Providers will be asked to include their email address at the end of both surveys as well as a unique digital identifier (i.e., the last two digits of their phone numbers and their two-digit birth month) to link the Time 1 and Time 2 surveys and to eliminate potential duplicates. Total time to complete the Time 2 survey is approximately 15 min. Participants who complete the Time 2 survey measures will be entered into another raffle for the chance to win a $25 gift card from a list of university-approved vendors. A total of 20 winners will be selected using a random number generator in Excel.

### Key constructs and measures

The following measures will be used to investigate the constructs proposed by the TPB (i.e., attitudes about screening for TBI, perceived behavioral control over TBI screening, subjective norms, intention to screening for TBI, and TBI screening behaviors) (Aim 1) and the constructs proposed by Diffusions of Innovations Theory (i.e., acceptability, feasibility, and appropriateness of TBI screening using the OSU TBI ID) (Aim 2). See Table 1.

**Table 1:** Measures, Key Constructs, and Definitions of Constructs

Construct	Definition	Aim	Measure	Timepoint	Theory	Variable
Attitudes	“The degree to which a person has a favorable or unfavorable evaluation or appraisal of the behavior in question.” [35]	1	TPBQ-TBI	1	TPB	Predictor
Perceived Behavioral Control	“Perception of the ease or difficulty of performing the behavior of interest.” [35]	1	TPBQ-TBI	1	TPB	Predictor
Subjective Norms	“The perceived social pressure to perform or not to perform the behavior.” [35]	1	TPBQ-TBI	1	TPB	Predictor
Adoption	“*Intention*, initial decision, or *action* to try or employ an innovation or evidenced-based practice” [3] and is sometimes referred to as “uptake” of an EBP.	1,2	TPBQ-TBI, Proportion of TBI screens conducted	1,2	TPB, DOI	Intention: Mediator (Aim 1) Predictor (Aim 2) Behavior: Primary Outcome
Acceptability	“The perception among implementation stakeholders that a given treatment, service, practice, or innovation is agreeable, palatable, or satisfactory.” [3]	2	AIM	2	DOI	Moderator
Feasibility	“The extent to which a new treatment, or an innovation, can be successfully used or carried out within a given agency or setting.” [3]	2	FIM	2	DOI	Moderator
Appropriateness	“The perceived fit, relevance, or compatibility of the innovation or evidence based practice for a given practice setting, provider, or consumer; and/or perceived fit of the innovation to address a particular issue or problem.” [3]	2	IAM	2	DOI	Moderator

#### Theory of planned behavior constructs

The 28-item TPB Questionnaire for TBI (TPBQ-TBI) will be used to measure provider attitudes, subjective norms, perceived behavioral control, and intentions to adopt the OSU TBI-ID. The TPBQ-TBI was adapted from a previously established TPBQ measure by tailoring the referent in each item (e.g., directing participants to refer to the OSU TBI-ID) [39]. A total of 24 items were retained from the original measure and 4 items were added and adapted from another TPBQ measure to capture items relevant to the present study but that were excluded from the original measure [47].

##### Attitudes

Thirteen items will be used to assess provider attitudes toward conducting TBI screening using the OSU TBI-ID. Three items assess attitudes regarding compatibility of the intervention; three items assess perceived ease of use of the intervention; four items assess perceived usefulness of the intervention; and three items assess overall attitudes toward the intervention. Each item is measured on a 7-point Likert scale ranging from 1 (*strongly disagree*) to 7 (*strongly agree*) and summed for a total score. Higher scores on the attitudes subscale reflect more positive attitudes toward screening for TBI using the OSU TBI-ID. The original TPBQ measure demonstrated high internal consistency reliability for attitudes (α = 0.94) [39].

##### Perceived behavioral control

Five items will be used to measure perceived behavioral control over using the OSU TBI-ID. Items are measured on a 7-point Likert scale ranging from 1 (*strongly disagree*) to 7 (*strongly agree*) and summed for a total score. Higher scores on this subscale equate to greater perceived control over TBI screening behaviors and self-efficacy. The original TPBQ measure demonstrated acceptable internal consistency reliability for perceived behavioral control (α = 0.77) [39].

##### Subjective norms

Five items will measure perceived social pressure to screen for TBI. Each item is measured on a 7-point Likert scale ranging from 1 (*strongly disagree*) to 7 (*strongly agree*) and summed for a total score. Higher scores on this subscale equate to more positive norms associated with TBI screening. The original TPBQ measure demonstrated good internal consistency reliability for subjective norms (α = 0.87) [39].

##### Intentions

Three items will be used to measure intentions to screen for TBI using the OSU TBI-ID over the following month. Items are measured on a 7-point Likert scale ranging from 1 (*strongly disagree*) to 7 (*strongly agree*) and summed for a total score. Higher scores equate to greater intentions to screen for TBI using the OSU TBI-ID. The original TPBQ measure demonstrated high internal consistency reliability for subjective norms (α = 0.92) [39].

##### TBI screening behaviors

TBI screening behaviors will be measured as a continuous, individual-level variable using 4-items aimed to capture the proportion of TBI screens conducted over a 1-month period relative to the number of clients who sought treatment during that period. TBI screening behaviors will be determined by the following questions: “Overall, how many *new* clients sought services from you over the last month?” “How many times did you screen for TBI using the OSU TBI-ID with *new* clients over the last month?” “Overall, how many *established* clients did you see over the last month?” “How many times did you screen for TBI using the OSU TBI-ID with *established* clients over the last month?” The total number of positive TBI screens will also be assessed by provider self-report of the number of individuals who screened positive for having a lifetime history of TBI.

##### Acceptability

The Acceptability of the Intervention Measure (AIM) will be used to measure acceptability of using the OSU TBI-ID in behavioral healthcare settings [48]. Each of the items will be adapted to replace “intervention” with intervention of interest for this study (e.g., OSU TBI-ID). This scale has 4-items which are measured on a 5-point Likert scale ranging from 1 (*completely disagree*) to 5 (*completely agree*). Items are summed for a total score, where higher scores indicate greater acceptability. The AIM has demonstrated high internal consistency (α = 0.85) and test-retest reliability (*r* = 0.80).

##### Feasibility

The Feasibility of the Intervention Measure (FIM) will be used to measure feasibility of using the OSU TBI-ID in behavioral healthcare settings [48]. Each item will be adapted to replace “intervention” with intervention of interest for this study (e.g., OSU TBI-ID). This scale has 4-items which are measured on a 5-point Likert scale ranging from 1 (*completely disagree*) to 5 (*completely agree*). Items are summed for a total score, where higher scores indicate greater acceptability. The FIM has demonstrated high internal consistency (α = 0.89) and test-retest reliability (*r* = 0.88).

##### Appropriateness

The Intervention Appropriateness Measure (IAM) will be used to measure appropriateness of TBI screening using the OSU TBI-ID [48], and each items will be adapted to replace “intervention” with the OSU TBI-ID intervention for this study. This scale has 4-items which are measured on a 5-point Likert scale ranging from 1 (*completely disagree*) to 5 (*completely agree*). Items are summed for a total score, where higher scores indicate greater acceptability. The IAM has demonstrated high internal consistency (α = 0.91) and test-retest reliability (*r* = 0.73).

### Analyses

Descriptive statistics will be used to describe provider and behavioral health organization characteristics in SPSS v27 [49]. For Aim 1, structural equation modeling (SEM) will be conducted in Mplus 8.5 [50]. ‘Attitudes,’ ‘perceived behavioral control,’ and ‘subjective norms’ will be exogenous variables hypothesized to have direct effects on the endogenous variable, ‘intention,’ and an indirect effect through intention on the endogenous variable, ‘TBI screening behavior.’ SEM permits the investigation of the direct and indirect effects of the constructs from the TPB on TBI screening behaviors, removes measurement error from the main constructs, and allows for proper handling of the ordinal nature of the data. Because the TPBQ-TBI items are measured using ordinal response options, the robust Weighted Least Squares Mean and Variance (WLSMV) estimator will be used [51]. Because TBI screening is conducted by individuals and does not necessarily depend on organizational policies or team-level procedures, the data will not be clustered within organizations, treatment teams, or any other entity.

For Aim 2, hierarchical multiple regression will be conducted using SPSS v27 [49]. The three exogenous variables (attitudes, norms, control) will be added to Block 1, intention added to Block 2, and the interaction terms (intention x acceptability, intention x feasibility, and intention x appropriateness) will be added to Block 3 to test the moderating effects of the three exogenous variables on the relationship between intention and TBI screening behaviors. Moderators will be added to block 3 one at a time and will be retained in the model only if they are statistically significant. Interaction effects will be graphed to facilitate interpretation.

#### Power calculation

Using the MacCallum et al. (1996) power and Root Mean Square Error of Approximation (RMSEA) specifications for determining sample sizes in SEM and the Preacher & Coffman (2004) sample size computation in Rweb, a total of *N* = 53 participants are needed to sufficiently power the model with an alpha level of *p* < .05, *df* = 408, power level of .80, and RMSEA_alternative_ = .06 [52, 53]. However, using standard conventions for sample sizes in SEM, the minimum analytic sample will be *N* = 200 [54].

#### Model fit

Model fit for the SEM will be evaluated using the following fit indices and cutoffs: χ^2^ > .05, the Comparative Fit Index (CFI, > .95), Tucker Lewis Index (TLI, > .95), Standardized Root Mean Square Residual (SRMR, < .80), and the point estimate and 90% CI of the Root Mean Square Error of Approximation (RMSEA, < .06) [55]. Depending on the normality of the data, Maximum Likelihood or robust Maximum Likelihood estimation will be used.

#### Missing data

Missing Values Analysis (MVA) will be conducted to determine percentage and patterns of missing data [56]. Little’s test of Missing Completely at Random (MCAR) will be used as one method to determine patterns of missing data [57]. Missing at Random (MAR) test will also be conducted in SPSS by creating dummy variables of missingness on each of the variables with missing data and evaluating the differences. If data are found to be MCAR or MAR (*p* < 0.05), hypothesis testing will proceed in Mplus. The Mplus uses full information maximum likelihood (FIML) for handling missing data and can be used if data are MCAR or MAR [58].

### PHASE II (qual)

#### Aim 3

Assess the contextual determinants to TBI screening adoption. We will investigate determinants to TBI screening adoption through qualitative, semi-structured interviews with a subset of behavioral health providers who participated in the quantitative survey.

### Researchers’ position statement

Our previous experience using qualitative phenomenology [59] to assess behavioral health provider practices in treating clients with co-occurring TBI and substance use disorders will guide Phase II of this mixed methods study [30]. Our previous work using deductive thematic analysis using constructs from the Consolidated Framework for Implementation Research (CFIR) will guide our analytical approach [60].

#### Procedures

##### Data collection and sample size

A total of *N* = 20 providers who completed both Time 1 and Time 2 survey measures will be purposively selected using non-random, maximum variation sampling, with providers selected based on demographic-level differences (e.g., state, practice type) and response convergence with the quantitative results [61]. This method is expected to include a variety of providers employed across behavioral healthcare settings so that variations in determinants to TBI screening can be assessed across contexts. A total of *N* = 20 providers is needed to reach saturation of the data using a phenomenological research approach [59]. Providers will be contacted directly by email using the emails they provided in the surveys in Phase I. To account for the national sample and subsequent location variations of each provider, all interviews will be conducted through Zoom videoconferencing software and audio-recorded with the participant’s permission. Interviews are anticipated to last between 30 and 45 min. Participants who complete the interview will receive a $30 gift card from a list of OSU-approved vendors.

### Measures

A semi-structured interview guide will be developed based on results from Phase I [45]. A semi-structured interview protocol approach creates consistency between interviews with a standardized set of questions while allowing for probing and follow-up questioning [62]. The interview questions will be structured to assess contextual determinants to TBI screening adoption in behavioral healthcare settings guided by the quantitative results and constructs from CFIR.

### Analysis

Interview data will be managed and analyzed using NVivo 12.0 [63]. Interviews will be transcribed verbatim upon interview completion using a professional transcription service. Deductive, thematic analysis will be conducted using sensitizing concepts from CFIR [1, 64, 65]. Two coders will independently familiarize themselves with the data by reading each transcript, taking notes, and creating an initial set of codes based on CFIR’s five domains (e.g., characteristics of individuals, intervention characteristics, inner setting, outer setting, and process). Coders will meet to discuss the initial set of codes, then re-review transcript data, and refine codes into main themes. The review/revision process will continue until no new themes have emerged. Themes will be developed to ensure internal homogeneity (i.e., codes within themes share common features) and external heterogeneity (i.e., themes are distinct). Supporting quotes for each theme will be used to represent the essence of each theme.

#### Rigor

Co-coding, a detailed audit trail, and peer debriefing will be used to ensure rigor and reproducibility of the results [66]. A reflexive journal will also be used to bracket the authors’ thoughts and opinions about the study process, including experiences in recruitment, data collection, and analyses [62].

### Data integration

**Fig. 2:**
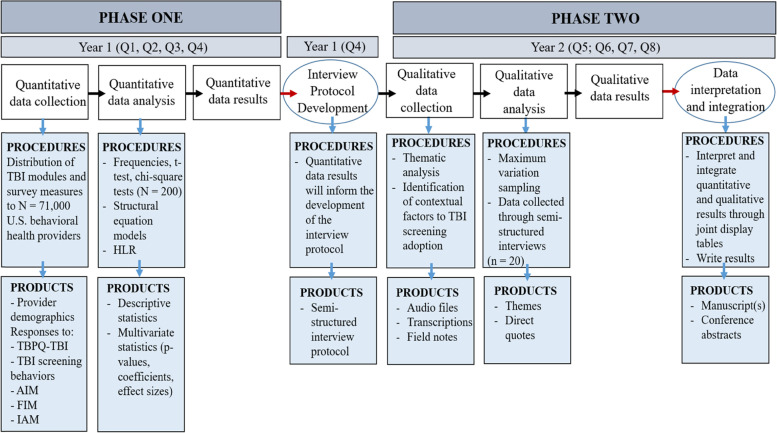
Procedural diagram for the explanatory sequential mixed methods design Note: Red arrows denote points of integration

To fully embody a mixed methods design, several points of data integration will occur [67, 68]. First, data from Phase I will be *connected* to Phase II by using the quantitative results to develop the qualitative interview protocol [69]. Second, the results from each phase will be *integrated* through a joint display table presenting both quantitative and qualitative data at the end of the entire study [70]. A joint display occurs at the reporting phase after all data have been collected and fully analyzed and is a visual method for presenting quantitative and qualitative results together. Following all data collection and analyses, the quantitative and qualitative data will be also integrated through *weaving* [67, 70]. Weaving occurs when results from both quantitative and qualitative data are written and presented in the text together on a concept-by-concept basis. A procedural diagram outlining the phases, steps, products, and timeline for this study is presented in Fig. 2 [43].

## Discussion

This project investigates provider-level characteristics, innovation-level factors, and contextual determinants to the early adoption of TBI screening in behavioral healthcare settings. Specifically, we will examine theory-driven relationships between characteristics of providers and the acceptability, feasibility, and appropriateness of the innovation by capitalizing on two hypotheses to bridge proximal (i.e., intention to screen) and distal outcomes (i.e., actual screening behavior). Testing these theory-driven hypotheses is necessary to understand the mediators, moderators, and causal pathways that lead to innovation adoption [8, 71]. This study moves the field of implementation science forward by testing these proposed hypotheses and identifying mediators and moderators that potentially influence the diffusion of a TBI screening innovation in behavioral health. In addition, by examining relationships among feasibility, acceptability, appropriateness, and screening adoption, we also have the potential to address questions about interactions among implementation outcomes [3]. Subsequently, implementation strategies that directly target these moderators and a causal chain of events can be developed, tested, and used in behavioral healthcare settings where TBI screening adoption is lagging*.*

Furthermore, because the behavioral health service context is broad and unique based on the specific setting, we will explore behavioral health providers perceptions about how the behavioral health context in which they work may affect TBI screening adoption. Our mixed methods approach could help us to better understand the complex interplay of this specific healthcare environment with the TBI screening innovation, particularly since TBI screening is new to this service context. Based on these results, implementation strategies can be identified that harness a social environment that is supportive of the innovation [72].

The expansion of literature over the past two decades has uncovered clear relationships between TBI and psychiatric comorbidities. However, few interventions have been developed specifically for individuals with these co-occurring conditions [73], and even fewer have penetrated the service landscape. This study is the first to take an existing TBI screening innovation used in physical health and community-based social service settings and brings it to the behavioral health treatment context using principles of implementation research and science. Behavioral health providers are a large segment of “untapped” professionals who could bridge gaps in service access by identifying individuals with co-occurring TBI and psychiatric comorbidities who may need additional services or adapted behavioral health treatment [30]. From here, TBI screening coupled with individualized adaptations to behavioral health delivery can be implemented to improve clinical care decisions and treatment options for individuals with co-occurring TBI and psychiatric comorbidities.

The potential benefits of this study are considerable to individuals who have co-occurring TBI, substance use disorders, and mental health comorbidities. Although TBI is one of the leading causes of death and disability in the United States, and TBI increases riskier substance use and mental health conditions, TBIs are often under recognized and under identified by behavioral health providers who are frequently treating individuals with these comorbidities [30]. Behavioral health providers who do not possess knowledge of TBIs or who do not intend to screen for TBI due to contextual factors will miss a large proportion of individuals in need of individualized treatment approaches and intervention decisions that account for the effects of their TBI. This study is the first to investigate the factors leading to the adoption of TBI screening in behavioral healthcare. Increasing TBI screening in behavioral health settings could have significant impact for how interventions and treatments are delivered, where and how referrals are made, and could reduce the risks for future injury or riskier substance use or worse mental health conditions associated with TBI.

## Supplementary Information

**Additional file 1.**

## Data Availability

Not applicable.

## Abbreviations

AIM: Acceptability of the Intervention Measure
CFI: Comparative Fit Index
CFIR: Consolidated Framework for Implementation Research
DOI: Diffusion of Innovations Theory
EBPs: Evidenced-based practices
FIM: Feasibility of the Intervention Measure
FIML: Full information maximum likelihood
IAM: Intervention Appropriateness Measure
JARS-MMR: Journal Article Reporting Standards for Mixed Methods Research
MCAR: Little’s test of Missing Completely at Random
MAR: Missing at Random
MVA: Missing Values Analysis
OSU TBI-ID: Ohio State University Traumatic Brain Injury Identification Method
RMSEA: Root Mean Square Error of Approximation
SRMR: Standardized Root Mean Square Residual
SEM: Structural equation modeling
TBI: Traumatic Brain Injury
TBP: Theory of Planned Behavior
TPBQ-TBI: Theory of Planned Behavior Questionnaire for TBI
TLI: Tucker Lewis Index
WLSMV: Weighted Least Squares Mean and Variance
