# Supplementary material for: Implementing traumatic brain injury screening in behavioral healthcare: protocol for a prospective mixed methods study
Source: Implement Sci Commun. 2022 Feb 14;3:17. doi: 10.1186/s43058-022-00261-x (PMC8842803; doi:10.1186/s43058-022-00261-x)
Supplement: Supplementary file 1 — Additional file 1. [file 43058_2022_261_MOESM1_ESM.docx]

| Table 2. | | | |
| --- | --- | --- | --- |
| *Mixed Methods Article Reporting Standards (MMARS): Information for Reporting the Collection and Integration of Qualitative and Quantitative Data (47)* | | | |
| Paper section or  element | Description of information to be reported | Recommendations for authors to consider & notes for reviewers | Location reported in article |
| Title page  Title | - See the JARS–Qual and JARS–Quant Standards. | - *Authors*: Refrain from using words that are either qualitative (e.g., *explore*, *understand*) or quantitative (e.g., *determinants*, *correlates*), because mixed methods stands in the middle between qualitative and quantitative research. - *Authors*: Reference the terms *mixed methods* or *qualitative and quantitative*. | ✓  (title page) |
| Author Note  Abstract | - See the JARS–Qual and JARS–Quant Standards. Abstract - See the JARS–Qual and JARS–Quant Standards. - Indicate the mixed methods design, including types of participants or data sources, and analytic strategy, main results/findings, and major implications/significance. | - *Authors*: Specify the type of mixed methods design used. See the note on types of designs in the methods research design overview section below. - *Authors*: Consider using one keyword that describes the type of mixed methods design and one that describes the problem addressed. - *Authors*: Describe your approach(es) to inquiry and, if relevant, how intersecting approaches to inquiry are combined when this description will facilitate the review process and intelligibility of your paper. If your work is not grounded in a specific approach(es) to inquiry or your approach would be too complicated to explain in the allotted word count, however, it would not be advisable to provide explication on this point in the abstract. | ✓  (abstract page) |
| Introduction  Description of research problems/  questions | - See the JARS–Qual and JARS–Quant Standards. | - *Authors*: This section may convey barriers in the literature that suggest a need for both qualitative and quantitative data. - *Reviewers*: Theory or conceptual framework use in mixed methods varies depending on the specific mixed methods design or procedures used. Theory may be used inductively or deductively (or both) in mixed methods research. | ✓  (pages 5 – 6; pages 9 - 12) |
|  |  |  |  |
|  |  |  |  |
| Table 2 *(continued)* | | | |
| Paper section or  element | Description of information to be reported | Recommendations for authors to consider & notes for reviewers | Reported in article |
| Study objectives/  aims/research  goals | - See the JARS–Qual and JARS–Quant Standards. - State three types of research objectives/aims/goals: qualitative, quantitative, and mixed methods. Order these goals to reflect the type of mixed methods design.   Describe the ways approaches to inquiry were combined, as it illuminates the *objectives* and mixed method rationale (e.g., descriptive, interpretive, feminist, psychoanalytic, postpositivist, constructivist, critical, postmodern or constructivist, or pragmatic approaches). | *Reviewers*: A mixed method objective, aim, or goal may not be familiar to reviewers. It describes the results to be obtained from using the mixed methods design type where “mixing” or integration occurs (e.g., the aim is to explain quantitative survey results with qualitative interviews in an explanatory sequential design). For instance, the goal of a qualitative phase could be the development of a conceptual model, the goal of a quantitative phase might be hypothesis testing based upon that model, and the goal of the mixed methods could be to generate integrated support for a theory based upon quantitative and qualitative evidence. | ✓  (page 10) |
| Method  Research design  overview | - See the JARS–Qual and JARS–Quant Standards. - Explain why mixed methods research is appropriate as a methodology given the paper’s goals. - Identify the type of mixed methods design used and define it. - Indicate the qualitative approach to inquiry and the quantitative approach used within the mixed methods design type (e.g., ethnography, randomized experiment) - If multiple approaches to inquiry were combined,   describe how this was done and provide a rationale (e.g., descriptive, interpretive, feminist, psychoanalytic, postpositivist, constructivist, critical, postmodern or constructivist, or pragmatic approaches), as it is illuminating for the mixed method in use.   - Provide a rationale or justification for the need to collect both qualitative and quantitative data and the added value of integrating the results (findings) from the two databases. | - *Reviewers*: Because mixed methods research is a relatively new   methodology, it is helpful to provide a definition of it from a  major reference in the field.   - *Reviewers*: Mixed methods research involves rigorous methods,   both qualitative and quantitative. Refer to the JARS–Qual standards (qualitative) and JARS–Quant standards (quantitative) for details of rigor.   - *Reviewers*: One of the most widely discussed topics in the mixed   methods literature would be research designs. There is not a  generic mixed methods design, but multiple types of designs. At  the heart of designs would be basic, core designs, such as a  convergent design, an explanatory sequential design, and an  exploratory sequential design. Although the names and types of  designs may differ among mixed methods writers, a common  understanding is that procedures for conducting a mixed methods study may differ from one project to another. Further, these basic procedures can be expanded by linking mixed methods to other designs (e.g., intervention or experimental trial mixed methods study), theories or standpoints (e.g., a feminist mixed methods study), or to other methodologies (e.g., a participatory action research mixed methods study). | ✓  (page 11) |
| Table 2 *(continued)* | | | |
| Paper section or  element | Description of information to be reported | Recommendations for authors to consider & notes for reviewers | Reported in article |
| Participants or other data sources | - See the JARS–Qual and JARS–Quant Standards. - When data are collected from multiple sources, clearly identify the sources of qualitative and quantitative data (e.g., participants, text), their characteristics, as well as the relationship between the data sets if there is one (e.g., an embedded design). - State the data sources in the order of procedures used in the design type (e.g., qualitative sources first in an exploratory sequential design followed by quantitative sources), if a sequenced design is used in the mixed methods study. | - *Authors*: Because of multiple sources of data collected, separate descriptions of samples are needed when they differ. A table of qualitative sources and quantitative sources is helpful. This table could include type of data, when it was collected, and from whom it was collected. This table might also include study aims/research questions for each data source and anticipated outcomes of the study. In mixed methods research, this table is often called an *implementation matrix*. - *Authors*: Rather than describe data as represented in numbers versus words, it is better to describe sources of data as open-ended information (e.g., qualitative interviews) and closed-ended information (e.g., quantitative instruments). | ✓  (page 14; See Figure 2. Procedural Diagram) |
| Researcher description | - See the JARS–Qual Standards. | - *Reviewers*: It is helpful to establish in a publication the researchers’ experiences (or research teams’ experiences) with both qualitative and quantitative research as a prerequisite for conducting mixed methods research. - *Authors*: Because mixed methods research includes qualitative research, and reflexivity is often included in qualitative research, we would recommend statements as to how the researchers’ backgrounds influence the research. | ✓  (page 21 and 23 on study rigor using reflexivity. |
| Participant  recruitment Participant  sampling or selection | - See the JARS–Qual and JARS–Quant Standards. - Describe the qualitative and the quantitative sampling in separate sections. - Relate the order of the sections to the procedures used in the mixed methods design type. |  | ✓  (PHASE I – page 14; PHASE II – page 21) |
| Participant recruitment | - See the JARS–Qual and JARS–Quant Standards. - Discuss the recruitment strategy for qualitative and quantitative research separately in mixed methods research. |  |  |
| Table 2 *(continued)* | | | |
| Paper section or  element | Description of information to be reported | Recommendations for authors to consider & notes for reviewers | Reported in article |
| Data collection Data collection/  identification procedures | - See the JARS–Qual and JARS–Quant Standards. | NA | ✓  (PHASE I – page 15; PHASE II – page 22) |
| Recording and transforming the data | - See the JARS–Qual Standards | NA | ✓  (page 23) |
| Data analysis | - See the JARS–Qual and JARS–Quant Standards. - Devote separate sections to the qualitative data analysis, the quantitative data analysis, and the mixed methods analysis. This mixed methods analysis consists of ways that the quantitative and qualitative results will be “mixed” or integrated according to the type of mixed methods design being used (e.g., merged in a convergent design, connected in explanatory sequential designs and in exploratory sequential designs). | NA | ✓  (PHASE I – page 20; PHASE II – page 23; Data integration – page 23; See Figure 2. Procedural Diagram) |
| Validity,  reliability, and methodological integrity | - See the JARS–Qual and JARS–Quant Standard Indicate methodological integrity, quantitative validity and reliability, and mixed methods validity or legitimacy. Further assessments of mixed methods integrity are also indicated to show the quality of the research process and the inferences drawn from the intersection of the quantitative and qualitative data. | NA | ✓  (PHASE I - pages 16 – 19; PHASE II – page 23) |
| Table 2 *(continued)* | | | |
| Paper section or  element | Description of information to be reported | Recommendations for authors to consider & notes for reviewers | Reported in article |
| Findings/Results Findings/Results  subsections | - See the JARS–Qual and JARS–Quant Standards. - Indicate how the qualitative and quantitative results were “mixed” or integrated (e.g., discussion, tables of joint displays, graphs, data transformation in which one form of data is transformed to the other, such as quantitative text, codes, themes are transformed into counts or variables). | - *Authors*: In mixed methods research, the findings section typically includes sections on qualitative findings, quantitative results, and mixed methods results. This section should mirror the type of mixed methods design in terms of sequence (i.e., whether quantitative strand or qualitative strand comes first; if both are gathered at the same time, either qualitative findings or quantitative results could be presented first). - *Reviewers*: In mixed methods Results sections (or in the Discussion section to follow), authors are conveying their mixed methods analysis through “joint display” tables or graphs that array the qualitative results next to the quantitative results (e.g., categorical or continuous data). This enables researchers to directly compare results or to see how results from the quantitative and qualitative strands. | Not applicable, however, a data integration plan is specified on page 23) |
| Discussion  Discussion  subsections | - See the JARS–Qual and JARS–Quant Standards | - *Authors*: Typically, the Discussion section, like the Method and Findings/Results, mirrors in sequence the procedures used in the type of mixed methods design. It also reflects upon the implications of the integrated findings from across the two methods | ✓  (page 24) |
| This table is adapted from Levitt HM, Bamberg M, Creswell JW, Frost DM, Josselson R, Suárez-Orozco C. Journal article reporting standards for qualitative primary, qualitative meta-analytic, and mixed methods research in psychology: The APA Publications and Communications Board task force report. *Am Psychol*. 2018;73(1):26. | | | |
|  |  |  |  |
|  |  |  |  |
